# *N*-(Quinoxalin-2-yl)-4-toluidine

**DOI:** 10.1107/S1600536808041160

**Published:** 2008-12-10

**Authors:** Wan Ainna Mardhiah Wan Saffiee, Azila Idris, Zaharah Aiyub, Zanariah Abdullah, Seik Weng Ng

**Affiliations:** a Department of Chemistry, University of Malaya, 50603 Kuala Lumpur, Malaysia

## Abstract

The aromatic and the aromatic fused-rings in the title compound, C_15_H_13_N_3_, open the angle at the planar N atom to 130.07 (13) and 129.98 (13)° in the two independent mol­ecules in the asymmetric unit. The amino N atom of one mol­ecule forms a hydrogen bond to the 4-N atom of an adjacent quinoxalinyl ring, generating a supra­molecular chain.

## Related literature

For the structure of *N*-(2-pyrid­yl)-4-toluidine, see: Fairuz *et al.* (2008 ▶); for that of *N*-(pyrazin-2-yl)-4-toluidine, see: Wan Saffiee *et al.* (2008 ▶). The title compound is isostructural with *N*-(quinoxalin-2-yl)-4-chloro­aniline; see: Idris *et al.* (2008 ▶).
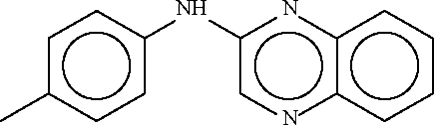


## Experimental

### 

#### Crystal data

C_15_H_13_N_3_
*M* *_r_* = 235.28
Orthorhombic, 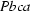

*a* = 12.2081 (9) Å
*b* = 11.3720 (9) Å
*c* = 35.097 (3) Å
*V* = 4872.5 (6) Å^3^
*Z* = 16
Mo *K*α radiation
μ = 0.08 mm^−1^
*T* = 100 (2) K
0.40 × 0.15 × 0.05 mm

#### Data collection

Bruker SMART APEX diffractometer
Absorption correction: none
26747 measured reflections
5592 independent reflections
4089 reflections with *I* > 2σ(*I*)
*R*_int_ = 0.051

#### Refinement

*R*[*F* ^2^ > 2σ(*F* ^2^)] = 0.041
*wR*(*F* ^2^) = 0.112
*S* = 1.03
5592 reflections
335 parameters
2 restraints
H atoms treated by a mixture of independent and constrained refinement
Δρ_max_ = 0.25 e Å^−3^
Δρ_min_ = −0.25 e Å^−3^

### 

Data collection: *APEX2* (Bruker, 2007 ▶); cell refinement: *SAINT* (Bruker, 2007 ▶); data reduction: *SAINT*; program(s) used to solve structure: *SHELXS97* (Sheldrick, 2008 ▶); program(s) used to refine structure: *SHELXL97* (Sheldrick, 2008 ▶); molecular graphics: *X-SEED* (Barbour, 2001 ▶); software used to prepare material for publication: *publCIF* (Westrip, 2009 ▶).

## Supplementary Material

Crystal structure: contains datablocks global, I. DOI: 10.1107/S1600536808041160/tk2339sup1.cif

Structure factors: contains datablocks I. DOI: 10.1107/S1600536808041160/tk2339Isup2.hkl

Additional supplementary materials:  crystallographic information; 3D view; checkCIF report

## Figures and Tables

**Table 1:** Hydrogen-bond geometry (Å, °)

*D*—H⋯*A*	*D*—H	H⋯*A*	*D*⋯*A*	*D*—H⋯*A*
N1—H1⋯N5	0.89 (1)	2.26 (1)	3.114 (2)	163 (2)
N4—H4⋯N2^i^	0.87 (1)	2.19 (1)	3.017 (2)	157 (2)

Symmetry code: (i) 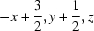.

**Fig. 1.**
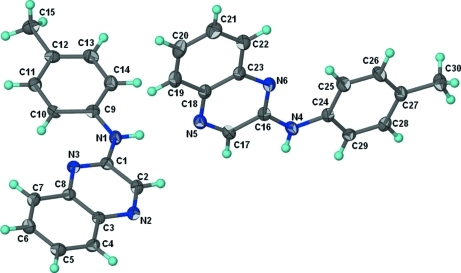
Thermal ellipsoid plot (Barbour, 2001) for the two independent molecules of C15H13N3 at the 70% probability level. Hydrogen atoms are drawn as spheres of arbitrary radius.

## supplementary crystallographic information

### Experimental

2-Chloroquinoxaline (1.64 g, 10 mmol) and 4-toluidine (1.07 g, 10 mmol) were mixed with ethanol (2 ml) and the mixture was heated at 423–433 K for 3 h. The product was dissolved in water and the solution extracted with ether. The ether phase was dried over sodium sulfate; the evaporation of the solvent gave well shaped crystals along with some unidentified brown material.

### Refinement

Carbon-bound H-atoms were placed in calculated positions (C—H 0.95 to 0.98 Å) and were included in the refinement in the riding model approximation, with *U*(H) set to 1.2 to 1.5_eq_*U*(C). The amino H-atoms were located in a difference Fourier map, and were refined with a distance restraint of N–H 0.88±0.01 Å; their temperature factors were freely refined.

### Crystal data

**Table:**

C_15_H_13_N_3_	*F*(000) = 1984
*M**_r_* = 235.28	*D*_x_ = 1.283 Mg m^−^^3^
Orthorhombic, *P**b**c**a*	Mo *K*α radiation, λ = 0.71073 Å
Hall symbol: -P 2ac 2ab	Cell parameters from 3585 reflections
*a* = 12.2081 (9) Å	θ = 2.5–27.6°
*b* = 11.3720 (9) Å	µ = 0.08 mm^−^^1^
*c* = 35.097 (3) Å	*T* = 100 K
*V* = 4872.5 (6) Å^3^	Block, yellow
*Z* = 16	0.40 × 0.15 × 0.05 mm

### Data collection

**Table:**

Bruker SMART APEX diffractometer	4089 reflections with *I* > 2σ(*I*)
Radiation source: fine-focus sealed tube	*R*_int_ = 0.051
graphite	θ_max_ = 27.5°, θ_min_ = 1.2°
ω scans	*h* = −15→15
26747 measured reflections	*k* = −14→9
5592 independent reflections	*l* = −45→45

### Refinement

**Table:**

Refinement on *F*^2^	Primary atom site location: structure-invariant direct methods
Least-squares matrix: full	Secondary atom site location: difference Fourier map
*R*[*F*^2^ > 2σ(*F*^2^)] = 0.041	Hydrogen site location: inferred from neighbouring sites
*wR*(*F*^2^) = 0.112	H atoms treated by a mixture of independent and constrained refinement
*S* = 1.03	*w* = 1/[σ^2^(*F*_o_^2^) + (0.0453*P*)^2^ + 2.027*P*] where *P* = (*F*_o_^2^ + 2*F*_c_^2^)/3
5592 reflections	(Δ/σ)_max_ = 0.001
335 parameters	Δρ_max_ = 0.25 e Å^−^^3^
2 restraints	Δρ_min_ = −0.25 e Å^−^^3^

### Fractional atomic coordinates and isotropic or equivalent isotropic displacement parameters (Å^2^)

**Table:**

	*x*	*y*	*z*	*U*_iso_*/*U*_eq_	
N1	0.48774 (11)	0.63997 (12)	0.55869 (4)	0.0200 (3)	
N2	0.54428 (10)	0.38803 (11)	0.61730 (3)	0.0183 (3)	
N3	0.41112 (10)	0.45078 (11)	0.55410 (3)	0.0169 (3)	
N4	0.78134 (10)	0.95678 (12)	0.67401 (4)	0.0194 (3)	
N5	0.57078 (11)	0.78948 (12)	0.62719 (4)	0.0207 (3)	
N6	0.59133 (10)	0.96931 (11)	0.68290 (3)	0.0183 (3)	
C1	0.47718 (12)	0.52591 (13)	0.57059 (4)	0.0166 (3)	
C2	0.54419 (12)	0.49311 (14)	0.60265 (4)	0.0188 (3)	
H2	0.5906	0.5510	0.6136	0.023*	
C3	0.47637 (12)	0.30629 (13)	0.60042 (4)	0.0164 (3)	
C4	0.47172 (12)	0.19136 (14)	0.61498 (4)	0.0193 (3)	
H4A	0.5162	0.1697	0.6361	0.023*	
C5	0.40277 (13)	0.11048 (14)	0.59870 (4)	0.0217 (3)	
H5	0.3993	0.0328	0.6086	0.026*	
C6	0.33726 (13)	0.14250 (14)	0.56743 (4)	0.0220 (3)	
H6	0.2897	0.0859	0.5563	0.026*	
C7	0.34082 (12)	0.25413 (14)	0.55266 (4)	0.0204 (3)	
H7	0.2964	0.2741	0.5314	0.024*	
C8	0.41034 (12)	0.33894 (13)	0.56900 (4)	0.0166 (3)	
C9	0.43642 (12)	0.69945 (14)	0.52834 (4)	0.0178 (3)	
C10	0.38087 (12)	0.64377 (14)	0.49874 (4)	0.0182 (3)	
H10	0.3741	0.5606	0.4983	0.022*	
C11	0.33549 (13)	0.71156 (14)	0.46985 (4)	0.0205 (3)	
H11	0.2981	0.6731	0.4497	0.025*	
C12	0.34273 (12)	0.83326 (14)	0.46935 (4)	0.0194 (3)	
C13	0.39785 (13)	0.88706 (14)	0.49930 (5)	0.0222 (3)	
H13	0.4036	0.9704	0.4999	0.027*	
C14	0.44450 (13)	0.82189 (14)	0.52828 (5)	0.0222 (3)	
H14	0.4823	0.8607	0.5483	0.027*	
C15	0.29166 (14)	0.90520 (15)	0.43798 (5)	0.0269 (4)	
H15A	0.3407	0.9702	0.4312	0.040*	
H15B	0.2213	0.9369	0.4467	0.040*	
H15C	0.2797	0.8553	0.4156	0.040*	
C16	0.67680 (13)	0.92171 (13)	0.66615 (4)	0.0174 (3)	
C17	0.66505 (13)	0.83080 (14)	0.63794 (4)	0.0198 (3)	
H17	0.7294	0.7994	0.6266	0.024*	
C18	0.47952 (13)	0.83502 (14)	0.64525 (4)	0.0190 (3)	
C19	0.37461 (14)	0.79237 (15)	0.63617 (5)	0.0247 (4)	
H19	0.3666	0.7318	0.6177	0.030*	
C20	0.28394 (14)	0.83771 (16)	0.65375 (5)	0.0274 (4)	
H20	0.2133	0.8084	0.6475	0.033*	
C21	0.29513 (13)	0.92756 (16)	0.68096 (5)	0.0265 (4)	
H21	0.2317	0.9591	0.6929	0.032*	
C22	0.39656 (13)	0.97021 (15)	0.69051 (4)	0.0226 (4)	
H22	0.4032	1.0306	0.7091	0.027*	
C23	0.49085 (12)	0.92445 (14)	0.67284 (4)	0.0178 (3)	
C24	0.81936 (13)	1.04794 (13)	0.69774 (4)	0.0177 (3)	
C25	0.75245 (13)	1.11936 (15)	0.72024 (4)	0.0233 (4)	
H25	0.6753	1.1083	0.7203	0.028*	
C26	0.79967 (13)	1.20656 (15)	0.74242 (5)	0.0242 (4)	
H26	0.7535	1.2550	0.7575	0.029*	
C27	0.91210 (13)	1.22577 (14)	0.74339 (4)	0.0202 (3)	
C28	0.97681 (12)	1.15459 (14)	0.72058 (4)	0.0191 (3)	
H28	1.0539	1.1662	0.7204	0.023*	
C29	0.93207 (12)	1.06744 (14)	0.69819 (4)	0.0180 (3)	
H29	0.9786	1.0200	0.6829	0.022*	
C30	0.96087 (14)	1.31961 (15)	0.76827 (5)	0.0263 (4)	
H30A	1.0407	1.3193	0.7654	0.040*	
H30B	0.9417	1.3042	0.7949	0.040*	
H30C	0.9320	1.3965	0.7607	0.040*	
H1	0.5235 (14)	0.6854 (15)	0.5750 (5)	0.038 (6)*	
H4	0.8327 (12)	0.9193 (14)	0.6617 (5)	0.027 (5)*	

### Atomic displacement parameters (Å^2^)

**Table:**

	*U*^11^	*U*^22^	*U*^33^	*U*^12^	*U*^13^	*U*^23^
N1	0.0240 (7)	0.0156 (7)	0.0204 (6)	−0.0025 (6)	−0.0051 (5)	0.0004 (5)
N2	0.0179 (6)	0.0195 (7)	0.0176 (6)	−0.0004 (5)	−0.0005 (5)	−0.0004 (5)
N3	0.0164 (6)	0.0162 (7)	0.0181 (6)	0.0003 (5)	0.0000 (5)	0.0003 (5)
N4	0.0166 (6)	0.0193 (7)	0.0223 (7)	−0.0015 (6)	0.0030 (5)	−0.0053 (5)
N5	0.0234 (7)	0.0180 (7)	0.0208 (6)	−0.0017 (6)	−0.0018 (5)	−0.0008 (5)
N6	0.0181 (6)	0.0189 (7)	0.0179 (6)	−0.0022 (5)	−0.0003 (5)	−0.0008 (5)
C1	0.0162 (7)	0.0163 (8)	0.0174 (7)	0.0000 (6)	0.0021 (6)	−0.0006 (6)
C2	0.0190 (7)	0.0195 (8)	0.0180 (7)	−0.0029 (6)	0.0000 (6)	−0.0020 (6)
C3	0.0147 (7)	0.0180 (8)	0.0164 (7)	0.0002 (6)	0.0024 (6)	−0.0010 (6)
C4	0.0198 (7)	0.0204 (8)	0.0177 (7)	0.0030 (6)	−0.0010 (6)	0.0023 (6)
C5	0.0244 (8)	0.0166 (8)	0.0240 (8)	0.0013 (7)	0.0004 (7)	0.0029 (7)
C6	0.0214 (8)	0.0187 (8)	0.0259 (8)	−0.0028 (7)	−0.0023 (6)	−0.0023 (7)
C7	0.0190 (8)	0.0210 (8)	0.0211 (7)	−0.0004 (7)	−0.0038 (6)	0.0001 (6)
C8	0.0152 (7)	0.0172 (8)	0.0174 (7)	0.0014 (6)	0.0015 (6)	0.0002 (6)
C9	0.0167 (7)	0.0184 (8)	0.0182 (7)	0.0003 (6)	0.0028 (6)	0.0011 (6)
C10	0.0205 (8)	0.0146 (8)	0.0194 (7)	0.0006 (6)	0.0032 (6)	−0.0017 (6)
C11	0.0219 (8)	0.0229 (9)	0.0167 (7)	0.0023 (7)	0.0009 (6)	−0.0016 (6)
C12	0.0183 (7)	0.0193 (8)	0.0206 (7)	0.0029 (6)	0.0045 (6)	0.0016 (6)
C13	0.0242 (8)	0.0151 (8)	0.0273 (8)	−0.0022 (6)	0.0021 (7)	0.0019 (7)
C14	0.0233 (8)	0.0197 (8)	0.0235 (8)	−0.0045 (7)	−0.0021 (6)	−0.0007 (7)
C15	0.0292 (9)	0.0241 (9)	0.0273 (9)	0.0034 (7)	−0.0026 (7)	0.0043 (7)
C16	0.0198 (7)	0.0155 (8)	0.0169 (7)	−0.0025 (6)	0.0003 (6)	0.0019 (6)
C17	0.0223 (8)	0.0177 (8)	0.0195 (7)	−0.0003 (7)	0.0012 (6)	−0.0019 (6)
C18	0.0225 (8)	0.0172 (8)	0.0175 (7)	−0.0023 (7)	−0.0026 (6)	0.0030 (6)
C19	0.0277 (9)	0.0235 (9)	0.0228 (8)	−0.0052 (7)	−0.0056 (7)	−0.0009 (7)
C20	0.0196 (8)	0.0334 (10)	0.0292 (9)	−0.0080 (7)	−0.0048 (7)	0.0012 (8)
C21	0.0199 (8)	0.0326 (10)	0.0270 (8)	−0.0012 (7)	0.0014 (7)	0.0003 (7)
C22	0.0218 (8)	0.0241 (9)	0.0219 (8)	−0.0012 (7)	0.0002 (6)	−0.0020 (7)
C23	0.0205 (8)	0.0171 (8)	0.0159 (7)	−0.0023 (6)	−0.0020 (6)	0.0027 (6)
C24	0.0211 (7)	0.0161 (8)	0.0159 (7)	−0.0018 (6)	−0.0009 (6)	0.0008 (6)
C25	0.0167 (7)	0.0251 (9)	0.0280 (8)	−0.0019 (7)	0.0019 (7)	−0.0059 (7)
C26	0.0220 (8)	0.0236 (9)	0.0269 (8)	−0.0008 (7)	0.0032 (7)	−0.0091 (7)
C27	0.0228 (8)	0.0184 (8)	0.0195 (7)	−0.0041 (6)	−0.0028 (6)	0.0009 (6)
C28	0.0169 (7)	0.0220 (8)	0.0184 (7)	−0.0028 (6)	−0.0018 (6)	0.0038 (6)
C29	0.0189 (7)	0.0183 (8)	0.0167 (7)	0.0029 (6)	0.0001 (6)	0.0011 (6)
C30	0.0252 (8)	0.0270 (9)	0.0269 (8)	−0.0060 (7)	−0.0014 (7)	−0.0060 (7)

### Geometric parameters (Å, °)

**Table:**

N1—C1	1.369 (2)	C12—C15	1.507 (2)
N1—C9	1.4090 (19)	C13—C14	1.381 (2)
N1—H1	0.886 (9)	C13—H13	0.9500
N2—C2	1.301 (2)	C14—H14	0.9500
N2—C3	1.3793 (19)	C15—H15A	0.9800
N3—C1	1.3096 (19)	C15—H15B	0.9800
N3—C8	1.3751 (19)	C15—H15C	0.9800
N4—C16	1.365 (2)	C16—C17	1.439 (2)
N4—C24	1.4084 (19)	C17—H17	0.9500
N4—H4	0.872 (9)	C18—C19	1.406 (2)
N5—C17	1.299 (2)	C18—C23	1.411 (2)
N5—C18	1.382 (2)	C19—C20	1.368 (2)
N6—C16	1.314 (2)	C19—H19	0.9500
N6—C23	1.3745 (19)	C20—C21	1.405 (2)
C1—C2	1.440 (2)	C20—H20	0.9500
C2—H2	0.9500	C21—C22	1.371 (2)
C3—C4	1.405 (2)	C21—H21	0.9500
C3—C8	1.415 (2)	C22—C23	1.407 (2)
C4—C5	1.372 (2)	C22—H22	0.9500
C4—H4A	0.9500	C24—C29	1.394 (2)
C5—C6	1.406 (2)	C24—C25	1.397 (2)
C5—H5	0.9500	C25—C26	1.386 (2)
C6—C7	1.372 (2)	C25—H25	0.9500
C6—H6	0.9500	C26—C27	1.390 (2)
C7—C8	1.407 (2)	C26—H26	0.9500
C7—H7	0.9500	C27—C28	1.386 (2)
C9—C10	1.393 (2)	C27—C30	1.502 (2)
C9—C14	1.396 (2)	C28—C29	1.378 (2)
C10—C11	1.389 (2)	C28—H28	0.9500
C10—H10	0.9500	C29—H29	0.9500
C11—C12	1.387 (2)	C30—H30A	0.9800
C11—H11	0.9500	C30—H30B	0.9800
C12—C13	1.390 (2)	C30—H30C	0.9800
			
C1—N1—C9	130.07 (13)	C12—C15—H15B	109.5
C1—N1—H1	113.7 (13)	H15A—C15—H15B	109.5
C9—N1—H1	115.3 (13)	C12—C15—H15C	109.5
C2—N2—C3	116.66 (13)	H15A—C15—H15C	109.5
C1—N3—C8	116.08 (13)	H15B—C15—H15C	109.5
C16—N4—C24	129.98 (13)	N6—C16—N4	122.10 (14)
C16—N4—H4	115.4 (12)	N6—C16—C17	121.65 (14)
C24—N4—H4	114.5 (12)	N4—C16—C17	116.24 (14)
C17—N5—C18	116.44 (13)	N5—C17—C16	123.24 (14)
C16—N6—C23	116.15 (13)	N5—C17—H17	118.4
N3—C1—N1	122.78 (14)	C16—C17—H17	118.4
N3—C1—C2	121.74 (14)	N5—C18—C19	120.06 (14)
N1—C1—C2	115.48 (13)	N5—C18—C23	120.37 (14)
N2—C2—C1	123.17 (14)	C19—C18—C23	119.57 (15)
N2—C2—H2	118.4	C20—C19—C18	120.31 (15)
C1—C2—H2	118.4	C20—C19—H19	119.8
N2—C3—C4	119.67 (13)	C18—C19—H19	119.8
N2—C3—C8	120.02 (14)	C19—C20—C21	120.13 (15)
C4—C3—C8	120.31 (14)	C19—C20—H20	119.9
C5—C4—C3	119.80 (14)	C21—C20—H20	119.9
C5—C4—H4A	120.1	C22—C21—C20	120.72 (16)
C3—C4—H4A	120.1	C22—C21—H21	119.6
C4—C5—C6	120.05 (15)	C20—C21—H21	119.6
C4—C5—H5	120.0	C21—C22—C23	120.01 (15)
C6—C5—H5	120.0	C21—C22—H22	120.0
C7—C6—C5	121.10 (15)	C23—C22—H22	120.0
C7—C6—H6	119.4	N6—C23—C22	118.66 (14)
C5—C6—H6	119.4	N6—C23—C18	122.09 (14)
C6—C7—C8	119.96 (14)	C22—C23—C18	119.25 (14)
C6—C7—H7	120.0	C29—C24—C25	118.58 (14)
C8—C7—H7	120.0	C29—C24—N4	116.69 (14)
N3—C8—C7	118.90 (13)	C25—C24—N4	124.72 (14)
N3—C8—C3	122.33 (14)	C26—C25—C24	119.36 (15)
C7—C8—C3	118.77 (14)	C26—C25—H25	120.3
C10—C9—C14	119.13 (14)	C24—C25—H25	120.3
C10—C9—N1	124.21 (14)	C25—C26—C27	122.46 (15)
C14—C9—N1	116.65 (14)	C25—C26—H26	118.8
C11—C10—C9	119.11 (14)	C27—C26—H26	118.8
C11—C10—H10	120.4	C28—C27—C26	117.18 (14)
C9—C10—H10	120.4	C28—C27—C30	121.66 (14)
C12—C11—C10	122.51 (15)	C26—C27—C30	121.15 (15)
C12—C11—H11	118.7	C29—C28—C27	121.58 (14)
C10—C11—H11	118.7	C29—C28—H28	119.2
C11—C12—C13	117.42 (14)	C27—C28—H28	119.2
C11—C12—C15	121.65 (15)	C28—C29—C24	120.82 (14)
C13—C12—C15	120.93 (15)	C28—C29—H29	119.6
C14—C13—C12	121.35 (15)	C24—C29—H29	119.6
C14—C13—H13	119.3	C27—C30—H30A	109.5
C12—C13—H13	119.3	C27—C30—H30B	109.5
C13—C14—C9	120.47 (15)	H30A—C30—H30B	109.5
C13—C14—H14	119.8	C27—C30—H30C	109.5
C9—C14—H14	119.8	H30A—C30—H30C	109.5
C12—C15—H15A	109.5	H30B—C30—H30C	109.5
			
C8—N3—C1—N1	179.27 (13)	C23—N6—C16—N4	−178.95 (14)
C8—N3—C1—C2	−0.5 (2)	C23—N6—C16—C17	2.1 (2)
C9—N1—C1—N3	−0.9 (2)	C24—N4—C16—N6	−4.0 (3)
C9—N1—C1—C2	178.94 (14)	C24—N4—C16—C17	174.97 (14)
C3—N2—C2—C1	0.4 (2)	C18—N5—C17—C16	−1.9 (2)
N3—C1—C2—N2	0.2 (2)	N6—C16—C17—N5	−0.2 (2)
N1—C1—C2—N2	−179.62 (14)	N4—C16—C17—N5	−179.18 (14)
C2—N2—C3—C4	−179.47 (14)	C17—N5—C18—C19	−177.95 (15)
C2—N2—C3—C8	−0.6 (2)	C17—N5—C18—C23	2.1 (2)
N2—C3—C4—C5	178.80 (14)	N5—C18—C19—C20	−179.52 (15)
C8—C3—C4—C5	−0.1 (2)	C23—C18—C19—C20	0.5 (2)
C3—C4—C5—C6	0.2 (2)	C18—C19—C20—C21	0.1 (3)
C4—C5—C6—C7	0.0 (2)	C19—C20—C21—C22	−0.6 (3)
C5—C6—C7—C8	−0.4 (2)	C20—C21—C22—C23	0.4 (3)
C1—N3—C8—C7	179.76 (13)	C16—N6—C23—C22	178.58 (14)
C1—N3—C8—C3	0.3 (2)	C16—N6—C23—C18	−1.9 (2)
C6—C7—C8—N3	−178.90 (14)	C21—C22—C23—N6	179.75 (15)
C6—C7—C8—C3	0.6 (2)	C21—C22—C23—C18	0.3 (2)
N2—C3—C8—N3	0.3 (2)	N5—C18—C23—N6	−0.2 (2)
C4—C3—C8—N3	179.13 (14)	C19—C18—C23—N6	179.87 (14)
N2—C3—C8—C7	−179.19 (13)	N5—C18—C23—C22	179.32 (14)
C4—C3—C8—C7	−0.3 (2)	C19—C18—C23—C22	−0.7 (2)
C1—N1—C9—C10	−15.1 (2)	C16—N4—C24—C29	−176.07 (15)
C1—N1—C9—C14	166.00 (15)	C16—N4—C24—C25	3.4 (3)
C14—C9—C10—C11	0.3 (2)	C29—C24—C25—C26	−0.4 (2)
N1—C9—C10—C11	−178.57 (14)	N4—C24—C25—C26	−179.90 (15)
C9—C10—C11—C12	−0.4 (2)	C24—C25—C26—C27	−0.3 (3)
C10—C11—C12—C13	−0.1 (2)	C25—C26—C27—C28	0.9 (2)
C10—C11—C12—C15	−179.21 (14)	C25—C26—C27—C30	−178.90 (15)
C11—C12—C13—C14	0.6 (2)	C26—C27—C28—C29	−0.8 (2)
C15—C12—C13—C14	179.69 (15)	C30—C27—C28—C29	179.05 (14)
C12—C13—C14—C9	−0.6 (2)	C27—C28—C29—C24	0.1 (2)
C10—C9—C14—C13	0.1 (2)	C25—C24—C29—C28	0.5 (2)
N1—C9—C14—C13	179.11 (14)	N4—C24—C29—C28	−179.91 (13)

### Hydrogen-bond geometry (Å, °)

**Table:**

*D*—H···*A*	*D*—H	H···*A*	*D*···*A*	*D*—H···*A*
N1—H1···N5	0.89 (1)	2.26 (1)	3.114 (2)	163 (2)
N4—H4···N2^i^	0.87 (1)	2.19 (1)	3.017 (2)	157 (2)

Symmetry codes: (i) −*x*+3/2, *y*+1/2, *z*.
